# A Study on the Association between *CCRΔ32* Mutation and HCV Infection in Iranian Patients

**Published:** 2018

**Authors:** Farahnaz Bineshian, Asieh Hosseini, Zohre Sharifi, Afsaneh Aghaie

**Affiliations:** 1. Department of Parasitology & Mycology, Faculty of Medicine, Semnan University of Medical Sciences, Semnan, Iran; 2. Blood Transfusion Research Center, High Institute for Research and Education in Transfusion Medicine, Tehran, Iran

**Keywords:** CCR5 protein, Hepatitic C, Human, Infection, Mutation

## Abstract

**Background:**

Mutations in the coding region of the Chemokine Receptor 5 (*CCR*5) genes reduce or eliminate *CCR5* expression in immune cells and progression of HCV infection. This study aimed to investigate the role of this mutation in HCV infection in Iranian patients in comparison with healthy individuals.

**Methods:**

100 HCV infected patients and 100 healthy individuals were randomly selected. The *CCR5Δ32* genotypes were determined using specific primers and PCR method.

**Results:**

The agarose gel electrophoresis showed a189-*bp* fragment from wild type for both alleles of *CCR5* gene. The *CCR5-Δ32* allele was not found in any HCV infected and healthy subjects.

**Conclusion:**

The mutation in *CCR5* gene was not detected in any of the two groups; therefore, the role of *CCR5* gene expression in immune cells and progression of HCV infection needs to be studied in larger samples in our country.

## Introduction

Hepatitis C is a complex liver disease and infection and Hepatitis C Virus (HCV) can cause acute and chronic liver disease throughout the world 1–3. Over the decades, the incidence of HCV has increased to 2.8% worldwide which results in more than 185 million infections 4,5. Also, Daw *et al* reported that HCV has been considered as a global threat in different geographical areas around the world 6. The prevalence of HCV infection is less than 1% of the general population in Iran 7.

HCV is known as a hepatotropic non cytopathic virus which can evade the immune response of the host. The effective immune response to HCV infection requires efficient recruitment and activation of inflammatory cells (monocytes and T lymphocytes) to the infected liver in which chemokines and chemokine receptors are involved and play important roles in the pathogenesis of chronic hepatitis C 8,9. CC-chemokines such as CCL3, CCL4, CCL5 and CCL3L1 bind to Chemokine (C-C motif) Receptor 5 (*CCR5*), thereby initiating activation and migration of cells. *CCR5* gene is located at position 3p21.31 on human chromosome. The coding sequence of this gene is 1056 base pairs, which is translated to a protein with 352 amino acid length 10. This protein is expressed by T helper lymphocytes type 1 (TH1), cytotoxic CD8 (+) T lymphocytes, monocytes, memory T cells (CD45RO), stem cells, dendritic cells, microglia and is known as an important co-receptor for macrophagetropic virus, including HIV-1, to enter host cells 10,11. The studies showed there are two promoter regions (an upstream promoter region and downstream promoter region), four exons (exon1, exon 2a/2b, exon 3) and two introns in the genomic organization of *CCR5* gene 11. A 32 *bp* deletion in the coding region of this gene, *CCR5Δ32*, leads to a frame shift in the open reading frame and produces a non-functional *CCR5* which is not expressed on the cells surface in homozygous patients (*CCR5Δ32/CCR5Δ32*) and its expression is decreased in heterozygous individuals (*CCR5Δ32*/wt) 10,12.

In a study on Caucasian population with homozygous subjects for *CCR5Δ32* allele, they were almost completely protected against Human Immunodeficiency Virus (HIV) infection, and heterozygous patients seem to have a delay in the progression of HIV 13. According to previous findings, *CCR5Δ32* role in HCV is not the same as HIV for entry into the cell. It is believed *CCR5Δ32* homozygosis may increase the risk of HCV infection 14,15. So far, there are no studies about association between *CCR5Δ32* mutation and HCV infection in Iranian patients. Therefore, this study aimed to investigate the role of this mutation in HCV infection in Iranian patients in comparison with healthy individuals.

## Materials and Methods

### Collection of samples

Blood buffy coat samples were collected from 100 HCV patients (age ranges between 15–81 years) and 100 healthy individuals (age range between 8–95 years). None of the participants had received antiviral therapy at the time of the study. All patients were positive for both anti-HCV antibody and serum HCV-RNA.

All specimens were randomly selected from people referred to clinical laboratory of the Iranian Blood Transfusion Organization in Tehran, Iran (IBTO). The protocol of this study was approved by the ethical committee of Blood Transfusion Research Center and the written informed consent was taken from both groups. Blood collection was performed in 10.8 *ml* K3 EDTA pre-coated glass tubes. After centrifuge of blood samples, buffy coat samples were stored at −80*°C* for further use.

### DNA extraction

Extraction of genomic DNA was performed by the salting out method described previously 16 from blood buffy coat samples. The extracted DNA Optical Density (OD) was obtained at wavelengths of 260 and 280 *nm* for being quantified by spectrophotometer instrument. Suitable DNA samples (OD_260_:OD_280_=1.8–2.0) were stored at −80*°C* for future use.

### PCR reaction

For detection of *CCR5* genotypes, specific primers from the previously published sequences were used (Table 1) 9. The PCR reaction was performed with a total volume of 25 *μl*, containing 12.5 *μl* 2× Master Mix (Takara), 0.5 *μl* in 10 *pmol* of each forward and reverse primers (CinnaGen) and 5 *μl* (1–100 *ng/μl*) extracted genomic DNA. The PCR program was done for 35 cycles on thermocycler (Corbett) as follows: denaturation at 98*°C*, 10 *s*; annealing at 56*°C*, 45 *s*; extension at 72*°C*, 45 *s*. Initial denaturation and final extension were at 98*°C* for 3 *min* and at 72*°C* for 10 *min*, respectively. Then, 10 *μl* of the amplified PCR products were electrophoresed on 1.5% agarose gel and stained with DNA green viewer to detect the presence and the proper size of PCR product. The wild type sample was obtained from the previous study as the negative control 17 and a synthesized gene in vector pGEM-B1 containing the *CCR5Δ32* gene (Bioneer Corp, Daejeon, South Korea) was used as the positive control. DNA molecular weight marker XIV (100 *bp* ladder from Roche) was also used to estimate the size of amplified DNA fragments on the gel.

**Table 1.** Primer sets

**Primer name**	**Primer sequences**	**Fragment length**
**CCR5 (Forward)**	5′-CAAAAAGAAGGTCTTCATTACACC-3′	189 *bp* (wild type)
**CCR5 (Reverse)**	5′-CCTGTGCCTCTTCTTCTCATTTCG-3′	157 *bp* (mutation type)

### Statistical analysis

Demographic data for the study of subjects were collected by questionnaire. All data were analyzed using the SPSS software for windows (Version 20.0). Chi-square test was used for statistical analysis. P- values<0.05 were considered significant.

## Results

**Figure 1.**
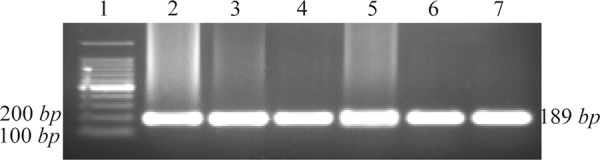
Agarose gel electrophoresis of PCR amplified DNA fragments for genotyping of CCR5 alleles. 1: 100 *bp* DNA ladder; 2-7: wild type (CCR5/CCR5).

The mean age of 200 participants in this research (132 males and 68 females) was 39.5±16 years. There was no significant difference between controls and cases in terms of the mean age (38.3 *vs*. 40.1, p=0.5). The characteristics of the study subjects in healthy and patient groups are compared in table 2. None of the gender ratio, age, level of education, ALT (Alanine aminotransferase) and AST (Aspartate aminotransferase) levels showed significant difference between two groups. The agarose gel electrophoresis showed 189-*bp* fragments from wild type for both alleles of *CCR5* gene (Figure 1). As shown in table 3, *CCR5-Δ32* allele was not found in any of the subjects.

**Table 2.** Demographic and clinical characteristics in HCV infected patients and healthy individuals

	**Healthy individuals**	**HCV patients**
**Number**	n=100	n=100
**Male/Female (%)**	61/39	71/29
**Age, years (Mean±SD)**	40.1±18.2	38.8±13.4
**Liver function test**		
**ALT *U/L* (Mean±SD)**	46±10.2	135.2±15.2
**AST *U/L* (Mean±SD)**	68.25±8.2	125.2±12.8

**Table 3.** Genotype frequencies of delta 32 CCR5 mutation in HCV infected patients and healthy individuals

**Subjects sample size**	**CCR5/CCR5 N(%)**	**CCR5Δ32/CCR5Δ32 N(%)**	**CCR5/CCR5Δ32 N(%)**
Anti-HCV- negative 100 healthy individuals	100 (100%)	0 (0)	0 (0)
Anti-HCV- positive 100 patients	100 (100%)	0 (0)	0 (0)

## Discussion

HCV is a major cause of acute and chronic hepatitis, cirrhosis and liver cancer worldwide. *CCR5* and its ligands by regulating the migration of immune cells to the infected liver play important role in the pathogenesis of hepatitis *C*. A 32-*bp* deletion in *CCR5* gene reduces or eliminates *CCR5* expression in immune cells and progression of HCV infection. Ahlenstiel *et al* reported that point mutation in the *CCR5-Delta32* interrupts the *CCR5* signaling pathway and reduces interferon gamma responses in anti-HCV positive haemophilic patients 18.

Morard *et al* studied a large cohort of 1,450 HCV infected patients and reported that 15.1% of heterozygous and 1.1% homozygous cases for CCRΔ32 alleles were associated with a decreased spontaneous HCV clearance 19. In the present study, homozygous (*CCR-5Δ32/CCR5Δ32*) or heterozygous (*CCR5/CCR5Δ32*) genotypes were not detected in any of HCV patients or healthy individuals. Therefore, the role of *CCR5* gene expression in immune cells and progression of HCV infection needs to be studies in larger samples in our country. Our results were consistent with some previous studies; for example, Khorram Khorshid *et al* reported that no Δ32/Δ32 genotype was detected among controls and Alzheimer’s patients 20. In another study on *CCR5* promoter polymorphism with chronic hepatitis C in Japan, *CCR5Δ32* mutation was not found in any of the 105 patients with chronic hepatitis C and 50 healthy individuals 9. In an Egyptian cohort of 150 anti-HCV positive chronic HCV patients and 100 healthy blood donors, the *CCR5Δ32* allele was not detected in the controls and only one case of homozygous genotype (*CCR5Δ32/ CCR5Δ32*) was seen in HCV patients 21. Also, El-Moamly *et al* showed that there was no association between the *CCRΔ32* mutation and HCV infection 22.

Several studies have been conducted in European countries and showed that the frequency of *CCR5Δ32* mutation decreased from northern to southern Europe and it is believed that this mutation first occurred in northern Europe 10. On the other hand, subjects homozygous for this mutation were reported to be approximately 1.1% in Caucasian population 23. So, the main reason for the difference in the results obtained on the influence of *CCR5Δ32* mutation on HCV infection can be various genetic makeup and race in different populations. In fact, since the patients with CCR5Δ32 were not detected in our study, the influence of *CCR5Δ32* as a genetic parameter upon susceptibility to HCV infection could not be assessed. There are different races in Iran such as Turk, Luri, Arab and historical evidence shows the genetic nature of Turk race in the North West of Iran which is similar to Caucasian people 24. Accordingly, our results must be treated with caution and it is recommended, due to the existence of various races in Iran, to perform more studies in larger samples or among different races in other regions, particularly the North West of Iran.

## Conclusion

Based on our findings, the mutation in *CCR5* gene was not detected in any of the two groups; therefore, the mutation in *CCR5* gene may be with low frequency in our country and the role of *CCR5* gene expression in immune cells and progression of HCV infection needs to be studies in larger samples in our country.
